# Mismatch repair deficiency may be common in ductal adenocarcinoma of the prostate

**DOI:** 10.18632/oncotarget.12697

**Published:** 2016-10-15

**Authors:** Michael T. Schweizer, Heather H. Cheng, Maria S. Tretiakova, Funda Vakar-Lopez, Nola Klemfuss, Eric Q. Konnick, Elahe A. Mostaghel, Peter S. Nelson, Evan Y. Yu, Bruce Montgomery, Lawrence D. True, Colin C. Pritchard

**Affiliations:** ^1^ Department of Medicine, Division of Oncology, University of Washington, Seattle, WA, USA; ^2^ Clinical Research Division, Fred Hutchinson Cancer Research Center, Seattle, WA, USA; ^3^ Department of Pathology, University of Washington, Seattle, WA, USA; ^4^ Division of Human Biology, Fred Hutchinson Cancer Research Center, Seattle, WA, USA; ^5^ Department of Laboratory Medicine, University of Washington, Seattle, WA, USA

**Keywords:** prostate cancer, ductal adenocarcinoma, hypermutation, mismatch repair, microsatellite instability

## Abstract

Precision oncology entails making treatment decisions based on a tumor's molecular characteristics. For prostate cancer, identifying clinically relevant molecular subgroups is challenging, as molecular profiling is not routine outside of academic centers. Since histologic variants of other cancers correlates with specific genomic alterations, we sought to determine if ductal adenocarcinoma of the prostate (dPC) – a rare and aggressive histopathologic variant – was associated with any recurrent actionable mutations. Tumors from 10 consecutive patients with known dPC were sequenced on a targeted next-generation DNA sequencing panel. The median age at diagnosis was 59 years (range, 40–73). Four (40%) patients had metastases upon presentation. Archival tissue from formalin-fixed paraffin-embedded prostate tissue samples from nine patients and a biopsy of a metastasis from one patient with castration-resistant prostate cancer were available for analysis. Nine of 10 samples had sufficient material for tumor sequencing. Four (40%) patients' tumors had a mismatch repair (MMR) gene alteration (*N* = 2, *MSH2*; *N* = 1, *MSH6*; and *N* = 1, *MLH1*), of which 3 (75%) had evidence of hypermutation. Sections of the primary carcinomas of three additional patients with known MMR gene alterations/hypermutation were histologically evaluated; two of these tumors had dPC. MMR mutations associated with hypermutation were common in our cohort of dPC patients. Since hypermutation may predict for response to immune checkpoint blockade, the presence of dPC may be a rapid means to enrich populations for further screening. Given our small sample size, these findings require replication.

## INTRODUCTION

Precision oncology entails therapeutic decision-making on the basis of an individual patient's molecular tumor profile. To that end, it is imperative to develop strategies to rapidly identify clinically relevant patient subgroups. While next-generation sequencing technologies have greatly advanced molecular classification, they are not routinely used for prostate cancer and may be costly. Because histological variants can correlate with genomic alterations in other malignancies (e.g. colorectal carcinoma, acute myelogenous leukemia), we hypothesized that distinct prostate cancer histologies may also associate with underlying molecular aberrations – allowing for the rapid identification of patients for further screening [1–5]. In this study, we sought to determine if ductal prostate cancer (dPC) was associated with clinically actionable molecular features.

Ductal prostatic adenocarcinomas (dPC) are an aggressive histopathologic variant of prostate cancer, characterized by large glands lined by tall, pseudostratified, columnar neoplastic epithelial cells [6]. Approximately 3% of all prostate cancers have at least a component of ductal histology, with only 0.2% having pure ductal histology [7]. Clinically, dPCs tend to have a more aggressive course – behaving similarly to Gleason 4 + 4 = 8 carcinomas [8]. Tumors with >10% ductal component are associated with a higher stage, are more likely to present with metastatic disease, and may be less responsive to androgen deprivation [7].

While the more aggressive clinical course associated with dPC has been well documented, little is known about the molecular features underlying this histologic subtype. Studies using fluorescence in situ hybridization have reported the prevalence of *TMPRSS2:ERG* fusions in ductal cases to range from approximately 10–50%, which is not substantially different than typical acinar carcinomas [9, 10]. Otherwise, gene expression profiling studies reveal extensive similarities between ductal and acinar adenocarcinomas. In one study comparing the transcriptional profile of eight ductal tumors to 11 acinar adenocarcinomas, differences in gene expression profiles encompassed only 25 genes [11].

Given that little is known regarding the underlying genomic abnormalities associated with the ductal histologic phenotype, we sequenced consecutive cases of dPC using the UW-OncoPlex platform – a targeted next-generation sequencing panel that includes genes with actionable or potentially actionable mutations [12].

## RESULTS

### Patient characteristics

**Figure 1:**
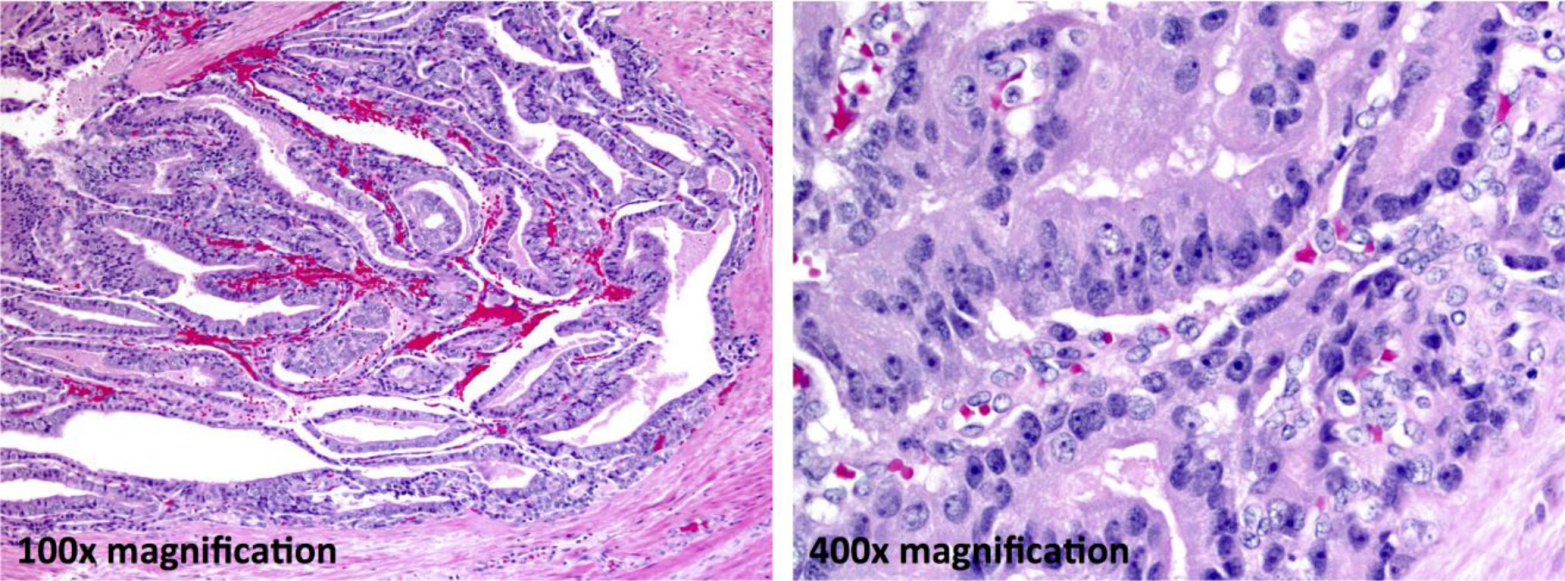
Ductal adenocarcinoma component In this case, approximately 65% of the carcinoma is ductal. Large tumor cell aggregates have a tubulopapillary architecture (100× final magnification). Forming a pseudostratified columnar epithelium the tumor cells have markedly atypical nuclei with clumped chromatin and prominent nucleoli (400× final magnification).

From January 2015 to April 2016, ten consecutive patients with dPC were identified and their tumors were sequenced (Figure 1). The median age at diagnosis was 59 years (range, 40 to 73). Four (40%) patients had metastatic disease at the time of presentation. Additional details regarding the patients included in this study and their tumor samples are provided in Table 1.

**Table 1:** Demographics

Subject number	Age at diagnosis	Gleason	Disease state at presentation	Disease state at Time of Tissue Acquisition	Source of tissue for UW-OncoPlex	Clinical state at last follow up	Time from diagnosis to last follow up (months)
1	72	9	Localized	Localized	Prostatectomy	NED	34.8
2	69	9	Metastatic	mHSPC	Needle Biopsy	mHSPC	8.2
3	52	8	Localized	Localized	Prostatectomy	NED	16.6
4	66	9	Localized	Localized	Prostatectomy	Death	10.3
5	73	7	Localized	Localized	Prostatectomy	Biochemical recurrence	28.1
6	51	8	Localized	Localized	Prostatectomy	NED	28.7
7	40	9	Metastatic	mCRPC	Prostatectomy	mHSPC	1.0
8	61	9	Metastatic	mHSPC	Needle Biopsy	mHSPC	29.6
9	58	9	Localized	mHSPC	Needle Biopsy	NED	15.3
10	54	7	Metastatic	Localized	Soft Tissue Met	mCRPC	16.5

mHSPC, metastatic hormone-sensitive prostate cancer; NED, no evidence of disease; mCRPC, metastatic castration-resistant prostate cancer.

### Sequencing results

To characterize the molecular features of dPC, we sequenced 10 prostate cancers with prominent dPC components: nine samples from FFPE archival tissue (radical prostatectomy or prostate needle biopsy specimens), and one frozen tissue biopsy from a metastasis. Nine of 10 samples had sufficient material for UW-OncoPlex testing. The tumors from four (40%) patients had an alteration predicted to be pathogenic in one of the mismatch repair (MMR) genes (2 in *MSH2*, 1 in *MSH6* and 1 in *MLH1*), of which 3 (75%) had evidence of hypermutation associated with microsatellite instability (MSI). The 3 patients with hypermutated tumors had evidence of bi-allelic MMR mutation. Other genomic alterations common to prostate cancer were also detected, including alterations in genes involved in homologous recombination repair (i.e. *BRCA2*, *CHEK2*) (*N* = 2), androgen receptor (*AR*) (*N* = 1), *TMPRSS2:ERG* rearrangements (*N* = 3) and alteration in the PI3K/Akt/mTOR signaling pathway (*N* = 5) (Table 2).

**Table 2:** Summary of somatic alterations identified in ductal prostate cancer cases

Subject number	Ductal component of sample used for NGS	Tumor content estimated from NGS	MMR gene alteration	HR gene alteration	Hypermutated	Total Coding Mutations (per 1.2Mb sequenced)	Selected Other Mutations and Variants
1	71%	30%	No	*CHEK2* c.1100delC+LOH	No	4	*PIK3CA* p.H1047Y, *PIK3R1* p.R577del, *CDH1* p.P373L, *EPHA5* p.R896H
2	45%	40%	*MSH2* inversion	No	No	4	*TP53* p.L252_I254del, *FOXA1* p.S304R
3	65%	60%	No	No	No	4	*TMPRSS2:ERG* rearrangement, *PTEN* p.F90Lfs*9 (only in 4% of reads), IKZF1 p.E35K, ABL2 c.347-1G>T, PML p.V452M, and *TRRAP* p.E1229Q
4	30%	60%	*MSH6* c.1900_1901del+LOH	No	Yes	29	*PTEN c*.968dup
5	97%	50%	*MSH2-GRHL2* rearrangement +LOH	No	Yes	34	(Many frameshift mutations attributable to MSI)
6	99%	50%	No	No	No	5	*IDH1*p.R132C, *CTNNB1* (beta catenin) p.S33A, and *FOXA1* p.M253_F254del
7	25%	0%	–	–	–	–	Insufficient tissue for sequencing
8	31%	70%	No	No	No	5	*PTEN* copy loss, *TMPRSS2:ERG* rearrangement, *TP53* p.E258G
9	35%	10%	No	*BRCA2* c.5946delT+likely LOH	No	3	*SPOP* p.D130E, *FLT1* (VEGFR) rearrangement
10	-	60%	*MLH1* exon 19+ 3′UTR homozygous deletion	No	Yes	32	*AR* p.W742L, *PIK3CA* p.H1047R, *TMPRSS2:ERG* rearrangement, *FOXA1* rearrangement

All mismatch repair (MMR) gene and homologous recombination (HR) gene alterations were known or predicted to be pathogenic. Note: metastatic tissue from subject 10 was sequenced. LOH, loss of heterozygosity; NGS, next generation sequencing.

### Histopathology of hypermutated prostate cancer

To determine the histopathologic features of hypermutated prostate cancer, we reviewed the pathology of known hypermutated cases from the University of Washington rapid autopsy program. We previously reported 5 prostate cancer patients who participated in this program and were found to have hypermutated tumors with complex MMR gene alterations [13]. Since that publication, we have identified 3 additional hypermutated prostate cancer cases using similar methods. Of the now 8 hypermutated prostate cancer cases in the autopsy series, 2 had untreated primary prostate cancer tissue available for pathology review. Both of these cases had a ductal adenocarcinoma component. The first subject (Autopsy Patient: 05-165) was previously reported to have an *MSH2-C2orf61* 343 kb inversion, *MSH2-KCNK12* 74 kb inversion, and *MSH2-KCNK12* 40 kb inversion [13]. The second subject (Autopsy Patient: 01-002), who was not included in our previous publication, had a germline *MSH2* exon 1–8 deletion with loss of heterozygosity in tumor tissue.

**Figure 2:**
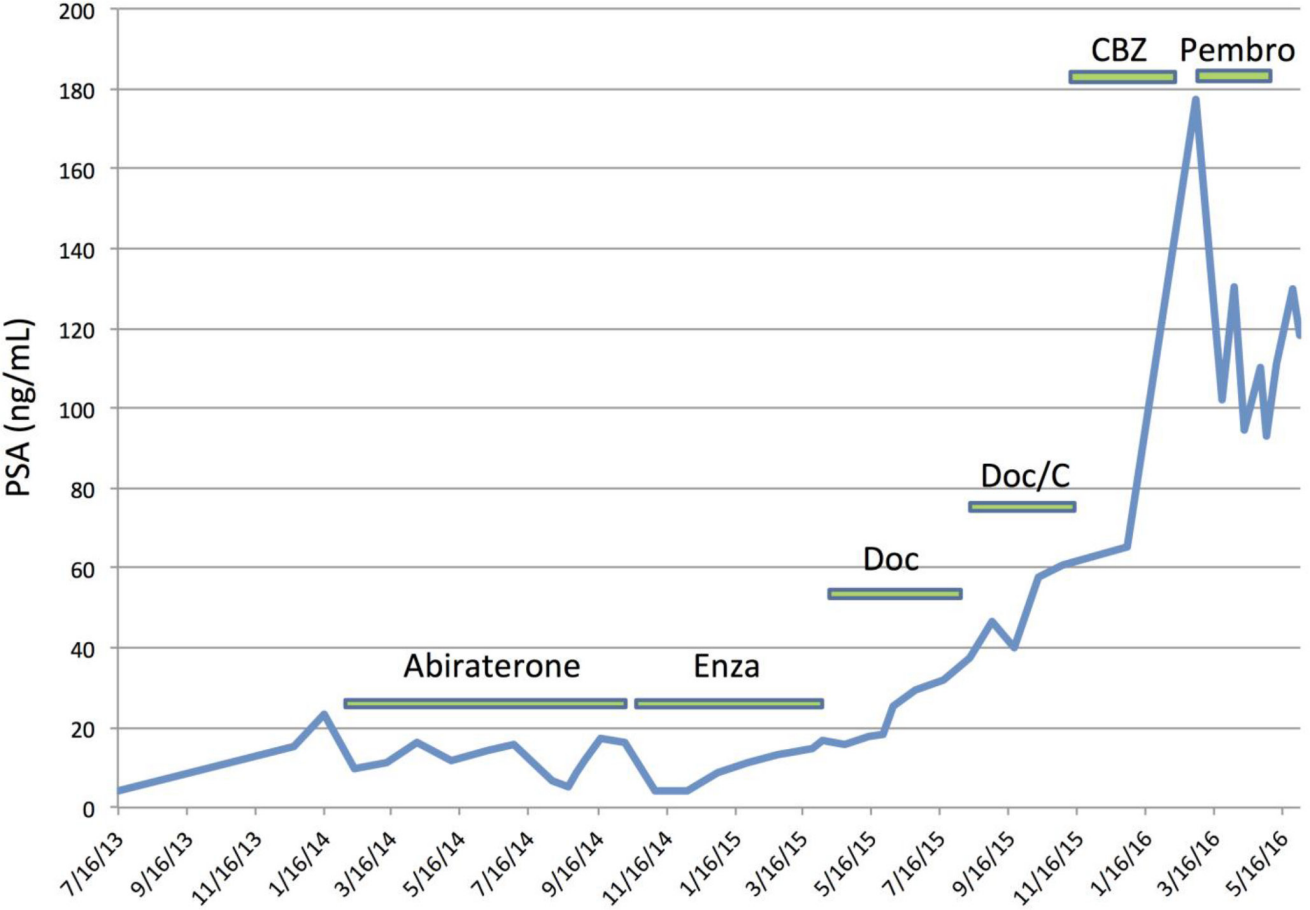
PSA response to checkpoint blockade immunotherapy in a patient with hypermutated prostate cancer Prior to initiating pembrolizumab, this patient had bone, adrenal and lymph node metastases, and a baseline PSA of 177.35 ng/mL. A total of 3 cycles of pembrolizumab were administered before stopping due to an immune related adverse event (anasarca) requiring corticosteroids. He expired in June 2016. Note: this patient did not have ductal histopathologic features. Enza, enzalutamide; Doc, docetaxel; C, carboplatin; CBZ, cabazitaxel; Pembro, pembrolizumab.

The tumor of a third patient with known hypermutated prostate cancer (determined through previously described methods) being followed in our clinic was histologically reviewed [14]. There was no ductal adenocarcinoma component in his tumor. It is notable, however, that this patient had a PSA decline following treatment with the immune checkpoint inhibitor pembrolizumab (i.e. anti-PD1) despite previously progressing on abiraterone, enzalutamide, docetaxel, carboplatin and cabazitaxel (Figure 2).

## DISCUSSION

This series of consecutive patients with dPC represents the largest next-generation sequencing study focused on this rare prostate cancer subset to date. Consistent with other published reports, patients in our series had aggressive clinical features, including young age at diagnosis and a high proportion of metastatic disease at presentation [6–8, 15]. Surprisingly, we found that alterations in MMR genes and associated hypermutation were far more prevalent in dPC compared to prostate cancers not selected by histologic subtype [13, 14]. Providing further support for an association between ductal histology and MMR deficiency, we found that two of three patients with MMR-deficient hypermutated metastatic prostate cancer whose primary tumors were available for review had dPC.

Hypermutated prostate cancers have only recently been described, with initial reported incidence ranging from approximately 3% to 12% in men with metastatic castration-resistant prostate cancer (mCRPC) [13, 14]. Although further validation to establish prevalence through larger systematic studies is needed, our findings are intriguing because they suggest a potential histologic association between the hypermutated genotype and a ductal histopathologic phenotype. More broadly, this finding supports an argument for sequencing rare histologic subtypes, as histology may provide insights into a tumor's underlying molecular features. Indeed, it is notable that a similar genotype-phenotype correlation in hypermutated MSI colorectal cancer has also been described – lending credence to the possibility that hypermutated cancers may have distinct histology compared to matched microsatellite stable cases [1, 3].

Determining which patients have hypermutated prostate tumors may have important implications for future precision oncology trials, as mutational burden has been shown to correlate with response to immune checkpoint blockade in several tumor types (e.g. anti-CTLA4, anti-PD1, anti-PDL1) [16–18]. Although objective responses to immune checkpoint inhibition have initially been generally disappointing in patients with prostate cancer, most have a relatively low mutational load [19, 20]. A recent Phase II study testing pembrolizumab (anti-PD1 therapy) in patients with metastatic colorectal carcinoma with and without MMR deficiency reported that 40% of hypermutated colorectal cancer patients had an immune-related objective response (irOR) compared to 0% of patients without MSI-high tumors. Moreover, a 50% response rate to pembrolizumab in hypermutated non-colorectal gastrointestinal malignancies has been observed – supporting the hypothesis that mutational load may be a predictive biomarker for response to immune checkpoint blockade in prostate cancer [17]. The observation that one of the hypermutated patients followed in our clinic had a dramatic response to anti-PD1 therapy in spite of being heavily pretreated further bolsters the hypothesis that hypermutation may be predictive of response to PD1/PDL1 pathway inhibition.

Consistent with our prior observations, we found that somatic loss-of-function mutations in *MSH2* and *MSH6* were the primary cause of microsatellite instability in patients with prostate cancer [13].This is in contrast to colorectal cancer where hypermutation has been found to be associated with epigenetic silencing of *MLH1*, which occurs in nearly 2/3 of the cases [21]. Interestingly, the tumor of Subject #2 showed evidence of *MSH2* inversion without clear evidence of hypermutation or MSI. Whether the *MSH2* loss-of-function alteration represents an early event and hypermutation is a later consequence in the disease course or follows selective pressures of treatment will need to be further examined. Given that the mechanisms underlying prostate cancer hypermutation appear distinct from colorectal cancer, patterns of MSI may also be divergent and a tailored approach to MSI testing of prostate cancer may be needed. However, our findings suggest that ductal histology may be a cue to investigate further for evidence of MMR deficiency and hypermutation.

The finding that prostate cancers with ductal histologic features may be enriched for somatic hypermutation is intriguing; however, our small sample size limits our ability to draw definitive conclusions regarding this genotype-histologic phenotype relationship. If this finding is confirmed, however, the presence of ductal adenocarcinoma histology could be a means to prioritize patients for additional studies to assess mutational burden, which may have clinical implications, as hypermutation appears to predict for response to immune checkpoint blockade in several cancer types, including early signals in prostate cancer [22]. Future efforts to define the landscape of genomic alterations in patients with this prostate cancer variant will likely require multi-institutional studies. Such studies may facilitate the promise and rapid completion of precision oncology approaches for targeting this molecular subset of prostate cancer.

## MATERIALS AND METHODS

### Patients

All patients carried a diagnosis of prostate cancer and were followed by a medical oncologist at the University of Washington Medical Center or Seattle Cancer Care Alliance (both in Seattle, Washington). Consecutive patients with a component of ductal adenocarcinoma were identified by the treating medical oncologist and offered tumor sequencing. After obtaining written informed consent, tumor samples were tested on the UW-OncoPlex platform [12].The original diagnoses of dPC, made by genitourinary (GU) pathologists (M.S.T., F.V.L.), were independently verified by a third GU pathologist (L.T.).

### Ethics statement

This study was performed in accordance with the declaration of Helsinki guidelines and with ethics approval from the Institutional Review Board at the Fred Hutchinson Cancer Research Center/University of Washington Comprehensive Cancer Consortium.

### Macrodissection of tumor tissue

Hematoxylin and eosin stained sections of the tumors were reviewed by an anatomic and molecular pathologist. Ten-micron unstained recut sections were cut from the FFPE block, which were determined to contain the maximum amount of ductal adenocarcinoma. The dPC component, which ranged from 20% to 99% of the cells by visual estimate of each tumor, was macrodissected prior to deparaffinization and DNA extraction.

### Next-generation sequencing (NGS) testing

DNA was extracted from FFPE samples as previously described [12]. Fresh tumor samples were snap frozen and unselected tissue was submitted for DNA extraction. UW-OncoPlex was performed according to previously published methods [12]. Microsatellite instability (MSI) testing was performed directly on NGS data using the mSINGS method [23]. Total mutation burden was estimated from targeted NGS data as previously described, with hypermutation defined as > 12 mutations/megabase [24].
